# Neural Progenitor Cell Terminology

**DOI:** 10.3389/fnana.2018.00104

**Published:** 2018-12-06

**Authors:** Verónica Martínez-Cerdeño, Stephen C. Noctor

**Affiliations:** ^1^Department of Pathology and Laboratory Medicine, Institute for Pediatric Regenerative Medicine and Shriners Hospitals for Children of Northern California, UC Davis School of Medicine, Sacramento, CA, United States; ^2^UC Davis Medical Center, MIND Institute, Sacramento, CA, United States; ^3^Department of Psychiatry and Behavioral Sciences, UC Davis School of Medicine, Sacramento, CA, United States

**Keywords:** stem cell, neural precursor cell, central nervous system, radial glial cell, intermediate progenitor cell, neurogenesis, proliferation, terminology

## Abstract

Since descriptions of neural precursor cells (NPCs) were published in the late 19th century, neuroanatomists have used a variety of terms to describe these cells, each term reflecting contemporary understanding of cellular characteristics and function. As the field gained knowledge through a combination of technical advance and individual insight, the terminology describing NPCs changed to incorporate new information. While there is a trend toward consensus and streamlining of terminology over time, to this day scientists use different terms for NPCs that reflect their field and perspective, i.e., terms arising from molecular, cellular, or anatomical sciences. Here we review past and current terminology used to refer to NPCs, including embryonic and adult precursor cells of the cerebral cortex and hippocampus.

## Introduction

“Stem cells” have the capacity to undergo self-renewing divisions that produce additional stem cells with the same properties and potential, and divisions that produce daughter cells that differentiate into multiple cell types. Stem cells can be “pluripotent precursor cells” that give rise to all cell types within an organism, or “multipotent precursor cells” that have the capacity to differentiate into a subset of cell types. The embryonic stem cells that are present in the inner cell mass of the blastocyst are an example of pluripotent stem cells. Many types of multipotent stem cells exist and can also be referred to as “progenitor cells.” The embryonic layers and each specific tissue, such as the central nervous system (CNS) tissue, develop from cellular divisions of progenitor cells.

“Neural progenitor cells (NPCs)” are the progenitor cells of the CNS that give rise to many, if not all, of the glial and neuronal cell types that populate the CNS. NPCs do not generate the non-neural cells that are also present in the CNS, such as immune system cells. NPCs are present in the CNS of developing embryos but are also found in the neonatal and mature adult brain, and therefore are not strictly embryonic stem cells. “Embryonic NPCs” may ultimately give rise to “adult NPCs,” as in the cerebral cortex (Merkle et al., 2004). NPCs are characterized based on their location in the brain, morphology, gene expression profile, temporal distribution and function. In general, embryonic NPCs have more potential than NPCs in the adult brain. NPCs can be generated *in vitro* by differentiating embryonic stem cells or “induced pluripotent stem cells (iPSC).” iPSCs are derived from adult cells, most often from fibroblasts or blood cells, and programmed into an embryonic-like pluripotent state.

## Embryonic Neural Progenitor Cells

Embryonic NPCs were first described in the fetal spinal cord by Camillo Golgi in 1885 (see Rakic, 2003). Neuroanatomists in the 19th century began to identify and characterize basic properties of NPCs and the proliferative zones in the developing brain (Kölliker, 1882; Magini, 1888; His, 1889; Lenhossek, 1891; Retzius, 1894; Schaper, 1897; Ramón y Cajal, 1911; Rakic, 2003). Work in the late 19th and early 20th century revealed mitotic cells dividing near the telencephalic ventricle and concluded these were the “germinal cells” that produced cortical neurons (His, 1889). Hamilton (1901) conducted what in our knowledge is the first developmental study of NPC distribution in the developing cortex. She plotted the location of mitotic precursor cells in the cerebral cortex and spinal cord at several stages of prenatal and postnatal development in the rat and showed that mitoses were positioned in two basic locations: at the lumen of the ventricle and away from the ventricle, which she termed “ventricular” and “extra-ventricular” mitoses (Hamilton, 1901). Hamilton found that there was a shift in the location of mitoses during development, with most precursor cells dividing at the ventricle during early stages of development, and the majority of precursor cells dividing away from the ventricle at later stages of development (Hamilton, 1901). In addition, Hamilton reported morphological differences among precursor cells that correlated with the position of the dividing cell—in other words that precursor cells at the ventricle and away from the ventricle were morphologically distinct (Hamilton, 1901).

### Embryonic Neural Proliferative Zones

Two proliferative zones in the developing cerebral cortex are commonly recognized today and using the terminology that was established in 1970 by the Boulder Committee (Angevine et al., 1970). The “ventricular zone (VZ)” is the primary proliferative zone that appears first during development and is adjacent to the ventricle, and the “subventricular zone (SVZ)” is the secondary proliferative zone that appears during later stages of development and is superficial to the VZ (Boulder Committee: Angevine et al., 1970). The only significant revision to Boulder Committee terminology in recent years stems from the work by Iain Smart and Henry Kennedy showing that the SVZ in rhesus monkeys is further subdivided into an “outer SVZ (oSVZ)” and an “inner SVZ (iSVZ)” (Smart et al., 2002). Subsequent work showed that the oSVZ is more prominent in the fetal human cortex (Fietz et al., 2010; Hansen et al., 2010), appears to be present in the developing cortex of most gyrencephalic mammals (Fietz et al., 2010; Reillo and Borrell, 2012), and is even present in the lissencephalic rat cortex during later stages of embryonic neurogenesis (Martínez-Cerdeño et al., 2012). The realization that the SVZ comprises distinct proliferative zones has stimulated significant lines of research into whether these different zones are populated by distinct NPC subtypes.

The terms that have been used to refer to NPCs in the developing cerebral cortex have varied over the past 100 years. These NPCs were initially referred to as “spongioblasts” and “fetal glia,” reflecting their presumed non-neuronal nature and non-mature glial cell morphology. The names of these cells changed over the course of time to reflect not only personal perspective but also appreciation of features that were newly revealed through application of new scientific technology. The morphology of NPCs in human and non-human primates were first characterized through whole-cell impregnation techniques such as Golgi staining, and were more fully characterized after the introduction of electron microscopy (Rakic, 1972) and immunohistochemistry (Levitt and Rakic, 1980). Because VZ cells in many species persist beyond birth and are arranged in a radial orientation in the telencephalon and other structures including the diencephalon and spinal cord, the combined term “radial glia (RG)” was introduced (Rakic, 1971a), and remains the most commonly used term for primary NPCs in the VZ.

### Embryonic Neural Progenitor Cells

RG cells appear through differentiation of precursor cells known as “neuroepithelial cells” that initially form the walls of the neural tube. Neuroepithelial precursor cells arise from the ectoderm early in development and are recognizable by their radial alignment and bipolar morphology—one process of the cell contacts the lumen of the ventricle, and the second process usually contacts the pial meninges. Neuroepithelial cells have the potential to undergo self-renewing symmetric divisions that increase the size of the precursor cell pool in early stages of development while forming the neural plate. After closure of the neural tube, neuroepithelial cells begin to upregulate glial specific factors, at which point they are thought to transform into RG cells and acquire the potential to generate neurons and glia (Aaku-Saraste et al., 1996; Morest and Silver, 2003). This cellular transformation is apparent at the morphological level by the lengthening of the cellular process that contacts the pial meninges, which we refer to as “pial fiber,” and which is also referred to as “basal process.”

RG cells located in the VZ are now considered to be the primary NPC in many regions of the developing brain. In the dorsal forebrain primary RG cells in the VZ can be identified by expression of the nuclear transcription factor Pax6 (Götz et al., 1998) and lack of expression for additional transcription factors such as Tbr2 (Englund et al., 2005). RG cells have been shown to exhibit several patterns of division and generate multiple cell types *in vitro* and *in vivo* during cortical histogenesis. RG cells initially undergo symmetric divisions that produce additional RG cells and expand the proliferative population in the VZ (Takahashi et al., 1995, 1996; Cai et al., 2002). *In vitro* experiments successfully replicate this feature of NPC behavior in the developing cerebral cortex (Noctor et al., 2008). At the onset of cortical neurogenesis, RG cells begin undergoing asymmetric divisions (Caviness et al., 2003), which produce a self-renewed RG cell and a neuronal daughter cell (Malatesta et al., 2000; Hartfuss et al., 2001; Miyata et al., 2001; Noctor et al., 2001; Tamamaki et al., 2001).

### Embryonic Neural Progenitor Cells in the Cerebral Cortex

**Figure 1:**
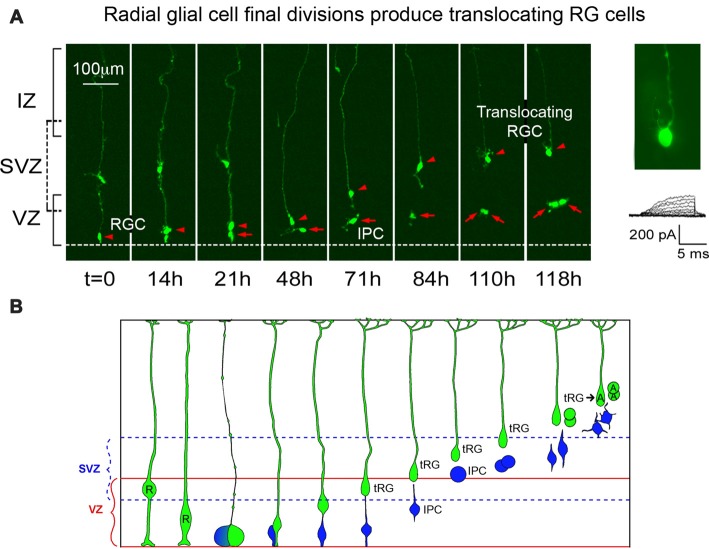
Adapted from Noctor et al. (2004) Nature Neuroscience with permission of Nature publishing group—Springer Nature. Radial glial (RG) cells divide at the surface of the ventricle to produce translocating RG (tRG) daughter cells. Panel **(A)** presents images from an organotypic slice culture prepared from embryonic rat. A time-lapse series began on E18 and showed a single RG cell (red arrowhead) that divided at the ventricle to produce a translocating daughter cell, which maintained the pial process (red arrowhead) and translocated toward the pia. A second daughter cell (red arrow) divided away from the surface (*t* = 110 h). Electrophysiological recording from the translocating cell at 118 h demonstrated an absence of the voltage-dependent inward current that is typical of astroglial cells. Panel **(B)** summarizes findings from multiple time-lapse series performed in embryonic rat. Following their final division at the ventricle, radial glial cells (R) translocate and begin transformation into astrocytes (A). One daughter cell is an intermediate progenitor (IP) cell (blue) that divides in the subventricular zone (SVZ). The tRG also continues dividing and electrophysiological recordings obtained from the newborn daughter cells show that they have the membrane properties of astroglial cells.

Later work demonstrated that asymmetric RG cell divisions appear to produce most neuronal daughter cells indirectly in the cerebral cortex, by first generating an NPC daughter cell that migrates to the SVZ, where it divides symmetrically to produce a pair of daughter neurons (Haubensak et al., 2004; Miyata et al., 2004; Noctor et al., 2004, 2008). Mitotic NPCs that divide in the SVZ have been identified by various terms, such as “extraventricular cells” (Hamilton, 1901), “subependymal cells” (Allen, 1912; Smart, 1961), “cells that divide away from the ventricle near blood vessels (BVs)” (Sauer, 1935), “SVZ cells” (Angevine et al., 1970), “non-surface progenitor cells” (Miyata et al., 2004), and some researchers have also used the term “abventricular mitoses.” Currently, two interchangeable terms are used for mitotic NPCs in the SVZ: “intermediate progenitor (IP) cells” (Noctor et al., 2004), and “basal progenitor cells” (Haubensak et al., 2004). IP cells are generally multipolar and can therefore be distinguished from bipolar RG cells based on morphology (Noctor et al., 2004, 2008). IP cells can often be distinguished from RG cells based on location of division and, in the cerebral cortex, by expression of the Tbr2 transcription factor (Englund et al., 2005). Evidence gathered to date from rodents suggests that cortical neurogenesis involves a series of amplifying divisions that can be characterized as a two-step process in which: (1) RG cells divide in the VZ to produce IP cells; and (2) IP cells divide in the SVZ to produce pairs of daughter neurons (Kriegstein et al., 2006; Martínez-Cerdeño et al., 2006). As a result of this pattern of division, each RG cell division produces two daughter neurons, and potentially more, depending on how many times each IP cell divides (Hansen et al., 2010). Similar patterns of amplifying divisions have been identified in the ventral forebrain (Lim and Alvarez-Buylla, 2014) and in the adult germinal niches (Seri et al., 2004; Figure 1).

In the 1970s evidence was presented on the detachment of RG cells from the ventricle and subsequent translocation toward the pial surface. The translocation of these cells is more frequent toward the end of the cortical neurogenic period, and their existence was hinted in early studies of the developing cortex that examined Golgi stained material (see Schmechel and Rakic, 1979). Translocating RG cells have been reported in fixed fetal tissue obtained from human (Choi and Lapham, 1978; deAzevedo et al., 2003), macaque (Schmechel and Rakic, 1979), ferret (Voigt, 1989) and rat (Noctor et al., 2004, 2008). As in the case of IP cells, multiple terms have been used to describe translocating RG cells, including “transitional RG” (Choi and Lapham, 1978; deAzevedo et al., 2003), “transitional astroglial cells” (Schmechel and Rakic, 1979), “transforming astroglial cells” (Voigt, 1989), “transforming RG cells” (Noctor et al., 2002), “translocating cells” (Noctor et al., 2004), “intermediate RG cells” (Reillo et al., 2011; Borrell and Reillo, 2012) and “translocating RG cells” (Martínez-Cerdeño et al., 2012). These cells are now referred to as “outer RG (oRG) cells” (Hansen et al., 2010) and “basal RG cells” (e.g., Fietz et al., 2010). *In vivo* and *in vitro* experiments in embryonic rat neocortex showed that translocating RG cells express glial fibrillary acidic protein (GFAP; Noctor et al., 2004), are mitotic, and generate glial cells (Noctor et al., 2008; Martínez-Cerdeño et al., 2012). More recent evidence shows that the translocating RG retain expression of the RG cell marker Pax6 (Fish et al., 2008; Fietz et al., 2010; Hansen et al., 2010; Reillo et al., 2011; Wang et al., 2011; Martínez-Cerdeño et al., 2012; Betizeau et al., 2013; Gertz et al., 2014; Poluch and Juliano, 2015), and may produce daughter neurons (Wang et al., 2011). Evidence gathered to date support the concept that translocating RG cells contribute to the population of astrocytes that are located in the cerebral cortex, including direct observations of *in vivo* data from sequential developmental stages (Voigt, 1989), live imaging of translocating cells followed by analysis through immunohistochemistry and electrophysiological recordings (Noctor et al., 2008), and data from early clonal lineage studies that provided evidence for mixed neuronal/astrocyte clones (Walsh and Cepko, 1990; Figure 1).

### Non-cortical Structures

Evidence suggests that at very early stages of development primary precursor cells across a number of CNS structures share fundamental characteristics. For example, mitotic precursor cells in the developing pineal gland express the nuclear transcription factor Pax6, express vimentin, undergo division at the ventricle and express phosphorylated vimentin during mitosis, as in the cortex (Ibañez-Rodriguez et al., 2016). In the pineal gland the primary precursor cells acquire distinct characteristics as development proceeds, at which point they no longer resemble cortical NPCs. These data support the idea of regional specialization of common NPC phenotypes that facilitate the generation of distinct cell types across the CNS.

### Human Brain

Nomenclature for NPCs in the human brain has largely been adopted from experimental animal models, in particular non-human primates (Smart et al., 2002). However, examination of developing human brain tissue has strengthened the case for unique features and characteristics of NPCs in human brain (Fietz et al., 2010; Hansen et al., 2010; Gertz et al., 2014; Otani et al., 2016). This may reflect functional adaptations of NPCs in the human brain that facilitate the production of more neurons and glia that are required to populate larger brain structures or may result from the evolution of functionally unique precursor cells that are not present in other mammals. Recent work examining single cell genomics of NPCs in the developing human brain will undoubtedly provide many answers for these questions (Nowakowski et al., 2017; Kosik and Nowakowski, 2018).

### Non-mammalian Vertebrates

Data from non-mammalian vertebrates, for example lizard, turtle and chicken points to NPCs that share features across a broad spectrum of vertebrates. For example, primary NPCs in the forebrain of developing lizards, turtles and chicken express Pax6 as in mammals. Furthermore, a dense band of Tbr2 cells is arranged in what appears to be an SVZ in the pallium of developing chick, and in the dorsal ventricular ridge of turtles (Martínez-Cerdeño et al., 2016). These data suggest that the evolution of NPC phenotypes is not recent or restricted to certain classes of mammals.

## Adult Neural Progenitor Cells

NPC are recognized as residing within two well-characterized niches in the adult mammalian brain: the “subgranular zone (SGZ)” of the dentate gyrus, and the “adult SVZ” surrounding the lateral ventricles of the mature cerebral cortex. The concept that neurons could be generated in the CNS of adult animals began with reports in the 1960s that neurons were generated in the postnatal rodent brain. Smart injected thymidine-H^3^ in 3-day old and adult mice, and found newborn neurons near the subependymal layer in neonatal mice (Smart, 1961). Smart also reported evidence of neuron production in the adult brain but did not find surviving neurons in the cerebral cortex and concluded that newborn neurons in the adult degenerated (Smart, 1961). Postnatal neurogenesis also takes place within the external granular layer (EGL) of the cerebellum. Precursor cells in these proliferative zones are derived from precursor cells in the prenatal brain. Sidman and colleagues showed that the cerebellar EGL arises from the embryonic cerebellar VZ/SVZ, and produces neurons during postnatal development (Miale and Sidman, 1961; Sidman and Rakic, 1973). Similarly, NPC in the dentate SGZ derive from the embryonic VZ (Nowakowski and Rakic, 1981). These data provide evidence that adult neural progenitor cells derive, at least in part, from embryonic precursor cells that seed the adult proliferative zones.

### Adult Neural Progenitor Cells in the Subgranular Zone

Adult NPCs in the dentate gyrus share fundamental properties with the RG cells and are therefore, referred to as “RG-like (RGL) cells” or “Type 1 cells.” Type 1 cells are located in the SGZ, have a complex radial process that extends through the granule cell layer to the molecular layer where its end-feet terminate on synapses and vasculature (Moss et al., 2016). Type 1 cells express nestin, GFAP, and Sox2, and generate adult granule neurons (Seri et al., 2001). Type 1 cells can be quiescent or proliferative, and when mitotically active can divide symmetrically and asymmetrically. During neurogenic divisions the Type 1 NPCs give rise to IP cells called “Type 2 cells” that, as in the developing cerebral cortex, express Tbr2, exhibit a multipolar morphology, and undergo a limited round of divisions that give rise to newborn neuronal cells that express doublecortin. The newborn daughter cells migrate radially into the granular cell layer where they mature into Prox1+ dentate granule neurons (Sun et al., 2015). Adult neurogenesis in the dentate gyrus has been observed in all mammals studied to date including humans (Eriksson et al., 1998; Ming and Song, 2011; Spalding et al., 2013; Hevner, 2016). The degree of adult neurogenesis in the dentate gyrus has been linked to crucial affective and cognitive behaviors, including learning, memory retention, pattern recognition and memory clearance (Sahay et al., 2011; Akers et al., 2014; Kitamura and Inokuchi, 2014; Anacker and Hen, 2017; for review see Berg et al., 2018).

### Neural Progenitor Cells in the Adult Subventricular Zone

**Figure 2:**
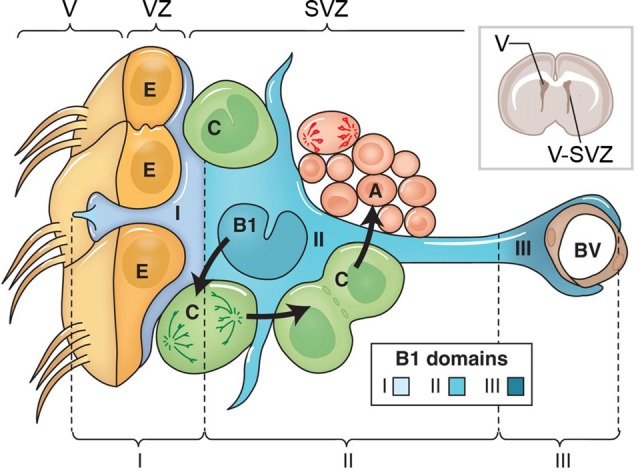
This figure adapted from Lim and Alvarez-Buylla (2016) with permission of Cold Spring Harbor Laboratory Press. The cellular composition of the ventricular zone (VZ) and SVZ that line the lateral ventricle (V) of the adult brain. The drawing at upper right shows the location of the lateral ventricle and the VZ and SVZ in a coronal section from an adult rat brain. The VZ and SVZ region is enlarged at the left. Type B1 cells (blue) are astrocytes that serve as the VZ/SVZ stem cell. B1 cells divide to produce Type C cells (green). C cells are rapidly dividing transit amplifying cells that produce to Type A cells (red), which are migratory neuroblasts. B1 cells contact blood vessels (BVs, brown). The apical surface of B1 cells makes contact with the ventricle and has a primary cilium. The apical surfaces of the B1 cells are found at the center of a “pinwheel” composed of multiciliated ependymal cells (Type E cells, yellow). The apical process of the B1 cells, and the multiciliated processes of the Type E ependymal cells extend from their respective cell bodies into the lateral ventricle. The boundary between the ventricle (V) and VZ is indicated by the brackets at top, and by the solid line in each Type E cell. The VZ/SVZ is subdivided into three domains: domain I contains the B1 cell apical processes and ependymal cells; domain II contains the cell body of B1 cells; and domain III contains the B1 cell contact with BVs.

New born cells generated in the adult cortical SVZ migrate rostrally to the olfactory bulb where they disperse and differentiate into interneurons (Figure 2). Adult NPCs in the SVZ also generate glial cells. Adult NPC are referred to as “B1 cells.” B1 cells are identified by location, expression of GFAP, GLAST and BLBP, and by endfeet that contact blood vessels (Doetsch et al., 1997; García-Verdugo et al., 1998). B1 cells can be in a quiescent or proliferative state. Proliferative B1 cells undergo asymmetric divisions to generate a self-renewed B1 cells and transient progenitor cells that acts as a transit amplifying cell known as “C cells” (Ortega et al., 2013). C cells subsequently undergo divisions that generate daughter cells referred as “A cells,” which migrate into the olfactory bulb. C cells express the transcription factors Ascl1 and Dlx2, while A cells express DCX and PSA-CAM (Doetsch et al., 1997; for review see Lim and Alvarez-Buylla, 2016).

### Adult Neural Progenitor Cells in the Third Ventricle, Fourth Ventricle, and Cerebral Aqueduct

Tanycytes are a subpopulation of ependymal cells that are located in the third ventricle surrounding the circumventricular organs. Tanycytes participate in the regulation of energy balance, energy homeostasis and chemosensitivity (Langlet et al., 2013; Langlet, 2014). Recent studies on the ultrastructural and molecular characterization of tanycytes in mouse identified these cells as E2 ependymal cells. E2 cells comprise a continuous epithelium along the floor of the cerebral aqueduct and fourth ventricle (Mirzadeh et al., 2017). Molecular properties of tanycytes in the third ventricle suggest that these cells are floor-plate derivatives (Mirzadeh et al., 2017). Proliferation of tanycytes located in the wall of the third ventricle is very limited under normal conditions in the adult rat brain, but can be induced *in vivo* (Mirzadeh et al., 2017; Hendrickson et al., 2018). More recently, it has been shown that the adult human hypothalamus contains four distinct populations of cells that express neuronal progenitor markers, each of these cell types, with the exception of tanycytes, are human-specific (Pellegrino et al., 2018).

### Adult Radial Glia Cells

Some RG cells in specific regions of the developing CNS differentiate into distinct RG cell types that persist into adulthood. These include “Müller glia” in the retina, “Bergmann glia (BG)” in the cerebellum (Guo et al., 2013; Surzenko et al., 2013), and RG cells in the adult spinal cord. BG derive from transformed RG cells and share many properties including radial alignment, multiple branching endfeet (Rakic, 1971b), vimentin expression, neuronal migration guidance (Schmechel and Rakic, 1979; Levitt and Rakic, 1980; Voigt, 1989), and mitotic activity (Bascó et al., 1977). Müller glia in the retina also derive from transformed RG cells and stain for vimentin and GFAP (Bignami, 1984; Pixley and de Vellis, 1984). In the adult retina, and specifically under conditions of stress, the RG derived Müller glia retain the potential to dedifferentiate, proliferate and generate newborn retinal neurons (Fischer and Reh, 2001).

## Conclusions

The variety of terms used by neuroscientists to describe NPCs have often reflected contemporary concepts arising from techniques that were prevalent at a given time to label, identify and view CNS cells. This is also true today to some degree. Research into CNS development has gained considerable knowledge in recent years through a combination of technical advances and individual insight. The terminology describing NPCs has evolved to incorporate newly revealed information about cellular characteristics and functions. While there has been a trend toward consensus and streamlined terminology, different terms for NPCs persist. The combined effect of research coming from diverse perspectives serves to increase our knowledge of what is ultimately most important: understanding NPC function in the developing and adult CNS.

## Author Contributions

Both authors wrote the manuscript.

## Conflict of Interest Statement

The authors declare that the research was conducted in the absence of any commercial or financial relationships that could be construed as a potential conflict of interest.
